# Measuring What Matters: Advancing Research on Intraoperative Pain During Cesarean Birth

**DOI:** 10.1111/aas.70335

**Published:** 2026-09-13

**Authors:** Silvia Marchesi, Sana Asif, Anne Juul Wikkelsø, Martin F. Bjurström

**Affiliations:** ^1^ Department of Clinical Science Malmö, Anesthesiology and Intensive Care Lund University Malmö Sweden; ^2^ Anaesthesiology and Intensive Care Medicine, Department of Surgical Sciences Uppsala University Uppsala Sweden; ^3^ Department of Anesthesiology and Intensive Care Zealand University Hospital Roskilde Denmark; ^4^ Department of Clinical Medicine University of Copenhagen Copenhagen Denmark; ^5^ Clinical Pain Research, Department of Surgical Sciences Uppsala University Uppsala Sweden

In 1990, Alahuhta and colleagues reported that approximately half of women undergoing cesarean section (CS) under neuraxial anesthesia experienced visceral pain; notably, sensory block height showed no significant association with this pain, challenging the assumption that higher blocks confer protection [1]. Decades later, pain during CS has overtaken accidental awareness under general anesthesia (GA) as the most common successful medico‐legal claim against British obstetric anesthetists [2].

Why does intraoperative pain during cesarean birth remain an unresolved problem despite advances in neuraxial anesthesia, and what is required to improve prevention, management, and follow‐up?

In this issue of the journal, a scoping review by Lund et al. brings these questions into sharp focus [3].

## 1 The Dimension of the Problem

Intraoperative pain affects between 8% and 23% of women undergoing CS [4, 5]. A precise European estimate is unavailable, as patient‐reported outcomes are not systematically collected, even in the largest prospective European multicenter study to date [6].

In a prospective multicenter cohort, 12% of women reported intraoperative pain; obstetricians failed to recognize it in 83% of cases and anesthesiologists in 52% [5]. The ACCESS study, comprising 9989 deliveries across 31 European countries, reported an overall GA rate of 15%, exceeding recommended targets at every urgency level. Strikingly, only 22% of these GAs were actually due to failed neuraxial block, suggesting that GA rates alone say little about how often blocks are truly inadequate [6].

The scoping review featured in this issue, synthesizing 63 studies, reveals a field that lacks even a shared language for what constitutes block failure: definitions range from objective sensory levels (variably tested) and pain NRS cut‐offs to supplementary analgesia requirements and conversion to GA. Even more tellingly, only two included studies addressed the patient's experience of inadequate blockade. The review confirms that our failure to define the problem is one of the problems.

In a randomized controlled trial comparing epidural top‐up with spinal anesthesia for CS, failure rates were 15% versus 3%, yet GA conversion rates were identical between groups, confirming that conversion to GA is a poor surrogate for neuraxial block failure [7].

Labor epidural augmentation carries the highest failure risk [8], yet in Scandinavian centers, epidural top‐up remains the primary anesthetic choice when a woman already has an epidural in place [9].

When intraoperative pain occurs, the ACCESS study showed that supplemental medication is administered in 21% of neuraxial cases, with 3% receiving sedatives specifically to avoid conversion to GA [6]. Midazolam is the most frequently administered agent [6], despite its hypnotic profile and lack of direct analgesic action. In Scandinavian practice, midazolam is used less frequently, but management remains largely guided by local preference and clinician experience rather than by standardized, evidence‐based protocols [10, 11].

## 2 What Do We Miss?

Central to this situation is one question: *How should we measure intraoperative pain during cesarean section?*

The most authoritative evidence converges on one point: conversion to GA is not a surrogate for intraoperative pain [5]. The French Practice Bulletin centers failure on the patient's request for additional medication [10, 12]; the Obstetric Anaesthetists' Association (OAA) on unsatisfactory surgical conditions and maternal comfort [13], irrespective of GA conversion [11]; and the American Society of Anesthesiology's 2024 Statement insists that the patient's experience must take precedence over the clinician's block assessment [14]. Yet conversion to GA remains the most commonly reported definition of neuraxial block failure.

The absence of a core outcome set is not merely theoretical; the numbers confirm it.

The wide variation in reported inadequacy reflects not true differences in incidence, but inconsistent definitions, or, in many cases, no patient input at all. In this context, Lund et al. do not simply document variability but expose a field that has systematically overlooked the very voice that should define the outcome.

It is remarkable that the only attempt at a guideline for the prevention and management of intra‐operative pain during CS comes from the dedication of a patient who, after experiencing a nightmare CS and subsequent post‐traumatic stress disorder (PTSD), pushed the medical community to take a stand. The guideline recommends, in case of intraoperative pain, using short‐acting intravenous opioids before delivery, as repeated boluses, with ketamine as an adjunct, and wound infiltration with local anesthetic after delivery [11]. Importantly, it stresses that when pain persists or is clearly present at block assessment, GA should be offered without delay.

The guideline also emphasizes non‐pharmacological elements: collection of truly informed consent, preceded by thorough explanation of the risk of pain; standardized neuraxial block assessment; communication with the patient throughout the procedure; and structured follow‐up for women who experience pain [11]. It surely provides a valuable foundation for the obstetric anesthesia community and has been followed by further documents endorsing this multilevel approach [15]. By considering the clinical, psychological, and relational dimensions of intraoperative pain, it reflects a broader understanding than a purely pharmacological approach would allow. Nevertheless, its existence also exposes the current state of the evidence: most recommendations are necessarily derived from expert consensus rather than high‐quality comparative studies. Moving from expert opinion to evidence‐based guidance will require standardized outcome measures, large multicenter trials, and systematic evaluation of interventions across the continuum of prevention, treatment, and follow‐up.

To broaden the perspective beyond managing pain once it occurs, it is imperative to recognize that intraoperative pain is not determined by anesthetic technique alone. Communication breakdown was reported as a contributing factor by nearly one in five respondents [16], and as the scoping review highlights, only 23% of anesthetists managing emergency CS report formal training in managing intraoperative pain [3].

Furthermore, the picture is never simply pain versus no pain. Maternal fear peaks at neuraxial block insertion, and negative prenatal expectations, birth partner anxiety, and perceived control over analgesia all influence perception [17]. Recalled pain and the feeling of having been dismissed by clinicians consistently exceed what was actually experienced, amplifying the risk of PTSD; 89% of women who suffered intraoperative pain during CS sustain moderate, and 11% permanent psychological sequelae [10, 11]. Structured psychological follow‐up is mandated by international guidelines [10, 14], yet remains absent from routine European practice.

## 3 What Do We Need?

The immediate priority is to establish and implement a *core outcome set* that enables consistent reporting across studies. Patient‐reported pain should serve as the primary outcome in multicenter studies, and quality of care should be anchored to this measure. But the outcome set must also include the multidimensional nature of intraoperative pain and discomfort during cesarean birth. Encouragingly, a growing body of qualitative work is informing this process by engaging directly with women affected by intraoperative pain and identifying the domains that should underpin a future core outcome set [18, 19].

Standardized outcomes would enable meaningful comparisons across studies, centers, and healthcare systems, overcoming the major barrier that has hindered progress in the field. Such harmonization would facilitate data pooling, meta‐analyses, and large multicenter trials aimed at identifying the most effective strategies for the prevention, recognition, and treatment of intraoperative pain.

Every woman reporting intraoperative pain should undergo postoperative review before discharge, including assessment for psychological sequelae such as post‐traumatic stress symptoms, and access to a clear referral pathway where appropriate [10, 11, 14]. A standardized core outcome set could also support the systematic collection of both immediate and longer‐term outcomes, generating the evidence base needed to inform routine practice and future recommendations.

By reading the work by Lund et al. [3], we are left with one uncomfortable truth: we have not agreed on what counts. Until the patient's experience takes precedence, we will keep citing the same numbers and failing the women who need us most.

## Author Contributions

All authors contributed equally to the literature review and the writing of the presenting editorial.

## Funding

The authors have nothing to report.

## Conflicts of Interest

The authors declare no conflicts of interest.

## Data Availability

Data sharing is not applicable to this article as no datasets were generated or analysed during the current study.
